# An investigation on [5 fluorouracil and epigallocatechin-3-gallate] complex activity on HT-29 cell death and its stability in gastrointestinal fluid

**DOI:** 10.18632/oncotarget.28207

**Published:** 2022-03-03

**Authors:** Laura Moracci, Francesca Sensi, Andrea Biccari, Sara Crotti, Elisa Gaio, Federico Benetti, Pietro Traldi, Salvatore Pucciarelli, Marco Agostini

**Affiliations:** ^1^Nano-Inspired Biomedicine Lab, Fondazione Istituto di Ricerca Pediatrica Città della Speranza, Padova, Italy; ^2^General Surgical Clinic 3, Department of Surgical, Oncological and Gastroentrological Sciences, University of Padua, Padova, Italy; ^3^Department of Molecular Sciences and Nanosystems, Ca’ Foscari University of Venice, Mestre, Venice, Italy; ^4^ECSIN-European Center for the Sustainable Impact of Nanotechnology, ECAMRICERT SRL, Padova, Italy

**Keywords:** HT29, 5-fluorouracil, epigallocatechin-3-gallate, molecular complexes, gastrointestinal model

## Abstract

Recently an enhancement of the sensitivity of colorectal cancer (CRC) cells by 5-fluorouracil (5FU) due to the concurrent treatment with epigallocatechin-3-gallate (EGCG) has been found. In the present paper, to investigate on this aspect, adenocarcinoma cells HT29 were treated with 5FU, EGCG and an equimolar mixture of 5FU and EGCG ([5FU+EGCG]) and cell viability was determined. While 5FU exhibits a clear activity, EGCG alone does not express any activity. However by treating the cells with [5FU+EGCG] a strong effect of EGCG is evidenced: the sensitivity of HT29 cells to 5FU was increased by 12-fold. A simulation of the behavior of [5FU+EGCG] in different compartments of the gastrointestinal digestion model was also performed. 5FU and EGCG solubilized into a mixture of digestive fluids analyzed by mass spectrometry did not lead to signals of 5FU, EGCG and the related complex, while by diluting the solution they become detectable. On the contrary, when 5FU and EGCG are submitted to the step-by-step digestion model procedure, the analysis did not show the presence of 5FU, EGCG and [5FU+EGCG]. This behaviour could be ascribed to the instability of these compounds due to the too severe digestion conditions and/or to the complexity of the matrix which could lead in ESI conditions to the suppression of the signals of the analytes of interest.

## INTRODUCTION

Colorectal cancer (CRC) is the fourth most prevalent cancer regarding incidence (6.1% of the total cases) and the second leading cancer for mortality (9.2% of the total cases) [1]. 5-fluorouracil (5FU)-based chemotherapy serves as the first-line, standard of care, chemotherapeutic treatment of choice in CRC patients.

After entering the cell, 5FU prevents cancer growth through converting to several active metabolites: Fluoro-deoxyuridine-monophosphate (FdUMP), Fluoro-deoxyuridine-triphosphate (FdUTP), and Fluoro-uridine-triphosphate (FUTP), which inhibit thymidylate synthase (TS) and block RNA and DNA synthesis [2]. However, in patients with advanced CRC the response rates to 5FU are merely 0–15% [3], and even combination treatments with oxaliplatin (FOLFOX) or irinotecan (FOLFIRI) inadequate responses are observed, the majority of the patients failing to respond to these treatments [4, 5]. Furthermore, the majority of chemotherapeutic drug failure in metastatic cancer has been attributed to *de novo* or *acquired* chemoresistance [6].

Numerous plant-based compounds exhibit chemotherapeutic activities [7–9], often combined with conventional treatments to reduce their secondary effects [10]. Natural polyphenolic phytochemicals, existing primarily in tea, have many clinical applications, among which anticancer agents [11]. In particular epigallocatechin-3-gallate (EGCG) exhibits numerous anti-inflammatory [12], antioxidant [13] and bone regulating properties [14, 15]. Numerous studies have demonstrated its chemopreventive and chemotherapeutic properties on cancer cell lines [16, 8, 10, 17]. EGCG sensitizes hepatocellular carcinoma cells to 5FU antitumor activity, and the combination of EGCG and 5-FU exhibits synergism in chemoresistant cancer cells [18]. Furthermore, it was shown that EGCG attenuates cell proliferation of oral squamous cell carcinoma cells by upregulation BTG2 expression via p38 and ERK pathways [19], that EGCG could target cancer stem-like cells and improve 5FU chemosensitivity in colorectal cancer [20] and that EGCG reverses 5FU resistance through TFAP2A/VEGF signalling inactivation, and MDR-1 and P-gp downregulation in gastric cancer [21]. Recently La et al. [22] demonstrated that EGCG enhanced the chemo-sensitivity of 5FU in low doses by inhibiting cancer proliferation, promoting apoptosis and DNA damage in CRC cells HCT-116 and DLD1. Mechanistically, EGCG blocks GRP78 expression, followed by enhancement of NF-κB and miR-155-5p levels, which further inhibits the MDR1 expression and promotes the 5FU accumulation in tumor cells. Collectively, this study implied that EGCG has a potential ability to serve as a chemo-sensitizer for traditional clinical drugs in colorectal cancer therapy. However, the role that EGCG could have as an adjuvant to both chemo- and radiotherapy for treating cancer from the mechanistic and biological points of view is still a topic of wide interest.

The results obtained by the above reported researches have been ascribed to the presence of synergies between 5FU and EGCG, i.e., by concurrent activities of the two molecules in the development of chemotherapeutic properties. Alternative to synergy, a different mechanism can be proposed, i.e., that based on the formation of non-covalent bimolecular complexes.

The formation of molecular complexes is a phenomenon widely observed in natural world [23] which can be considered the basis of “chemical evolution” [24]. In the case of natural substrates, consisting of hundreds (or thousands) of different molecular species in the condensed phase, the frequency of molecular interactions is very high. Thus, a natural substrate should not be considered as a set of isolated molecules but as an entity consisting of species generated from continuous processes of molecular interaction, in a situation of equilibrium dependent on thermodynamic conditions, following the rules of supramolecular chemistry [25]. As an example, in the case of caffeine, its complexation with polyphenolic substrates has been found and described in detail [26–28].

Considering the capability of catechins to form bimolecular non-covalent complexes, the interaction of catechins and 5FU was recently studied by different mass spectrometric approaches [29]. It was found that 5FU and catechins interact, leading to the formation of non-covalent complexes. Interestingly these results show that complexes not only with EGCG, but with other catechins, present in green tea extracts (GTEs) at lower concentration, are present. These molecular species, differently to free 5FU drug alone, would in principle possess a new biological activity and could be an explanation of the activity cited above originating by the presence of [5FU+catechin] non-covalent complexes.

Further studies are needed to uncover the molecular or chemical mechanisms by which EGCG is capable to enhance the 5FU activity and the present study is devoted to the comparison of the effects of 5FU, EGCG and of the mixture [5FU+EGCG] on human colorectal carcinoma HT29 cells, by analyzing cell viability and by examining the anti-proliferative effects. The main objective was to evaluate whether the combination of low doses of 5FU with EGCG could be equally or more effective than higher doses of 5FU alone and if, in this contest, the possible [5FU-EGCG] complex plays some possible roles.

Another aspect must be investigated, i.e., that related to the survival of 5FU, EGCG and of the [5FU+EGCG] complex in the gastro intestinal digestion conditions. Orally administered therapeutic agents must exhibit gastrointestinal stability to undergo efficient uptake. Solubility, permeability, stability, and metabolic interconversions may constrain the efficacy of dietary polyphenols, considering that different polyphenols exhibit different bioavailabilities [30–32]. It has been shown that their metabolic products may be more bioavailable than the parent compounds [33, 34]. The low bioavailability of dietary polyphenols may be due in part to their lability under conditions of the mammalian digestive tract. For example, EGCG undergoes oxidation and rearrangement reactions at neutral to basic pH and in the presence of dissolved oxygen [35, 36]. Although EGCG is attractive therapeutic agent because of their specific molecular mechanisms of action, and their natural occurrence in foods and traditional medicines, their probable low stability in gastrointestinal conditions could limit their utility. To investigate on this aspect a model simulating the human digestion in the oral, gastric and intestinal compartments with salt and protein composition, pH differences and transit times alike the *in vivo* digestion [37] can be employed. The model has been used so far for determining the bioaccessibility of orally ingested compounds like heavy metals [38] or other contaminants [39], and silica nano particles [40]. The constituents and concentrations of the digestive juices employed in the digestion model were described by Versantvoort et al. [39] and the related compositions are listed in Table 1. As can be observed, the different digestive juices are complex mixtures of organic compounds and inorganic salts and each of them is characterized by a well defined pH value. Consequently it would be to expect some difficulties in the direct analysis in ESI/MS conditions of juices containing 5FU and EGCG for the presence of the well described ion suppression phenomena. To overcome this aspect methods able to increase the specificity of the measurements are required and those achievable by tandem mass spectrometry (MS/MS) experiments have been tested for this aim.

**Table 1:** Composition of juices employed for the *in vitro* digestion model [39]

Saliva (pH 6.8 ± 0.1)	Gastric juice (pH 1.3 ± 0.1)	Duodenal juice (pH 8.1 ± 0.1)	Bile juice (pH 8.2 ± 0.1)	Sodium carbonate solution
896 mg KCl	2752 mg NaCl	7012 mg NaCl	5259 mg NaCl	84.7 g NaHCO3
200 mg KSCN	306 mg NaH2PO4 H2O	3388 mg NaHCO3	5785 mg NaHCO3	Milli-Q water
1021 mg NaH2PO4 H2O	824 mg KCl	80 mg KH2PO4	376 mg KCl	
570 mg Na2SO4	302 mg CaCl2	564 mg KCl	150 ml HCl (37%)	
298 mg NaCl	306 mg NH4Cl	50 mg MgCl2 6H2O	250 mg urea	
1694 mg NaHCO3	6.5 ml 37% HCl	180 ml HCl (37%)	167.5 mg CaCl2	
200 mg urea	650 mg glucose	100 mg urea	1.8 g BSA^*^	
290 mg amylase^*^	20 mg glucuronic acid	151 mg CaCl2	30 g bile^*^	
15 mg uric acid	85 mg urea	1 g BSA^*^	Milli-Q water	
25 mg mucin^*^	330 mg glucosaminehydrochloride	9 g pancreatin^*^		
Milli-Q water	1 g BSA^*^	1.5 g lipase^*^		
	2.5 g pepsin^*^	Milli-Q water		
	3 g mucin^*^			
	Milli-Q water			

^*^Protein components included in the “digestion with proteins” but not included in the “digestion without proteins”.

## RESULTS

### Investigation on the roles of 5 fluorouracil and epigallocatechin-3-gallate on HT-29 cell death

Samples of 5FU, EGCG and the equimolar mixture of 5FU+EGCG were injected in the LC/ESI/MS instrument (qTOF) operating in the conditions reported in the experimental section (negative ion detection). Different concentrations of 5FU and EGCG (1ng/μL and 10ng/μL) were considered and in both cases valid chromatographic and mass spectrometric data were obtained. The total ion chromatograms obtained by injection of 1 ng/μL solution of [5FU+EGCG] shows the presence of abundant [M-H]^−^ species of 5FU and EGCG, but any signal related to the complex ([5FU+EGCG-H]^−^) was undetectable.

**Figure 1:**
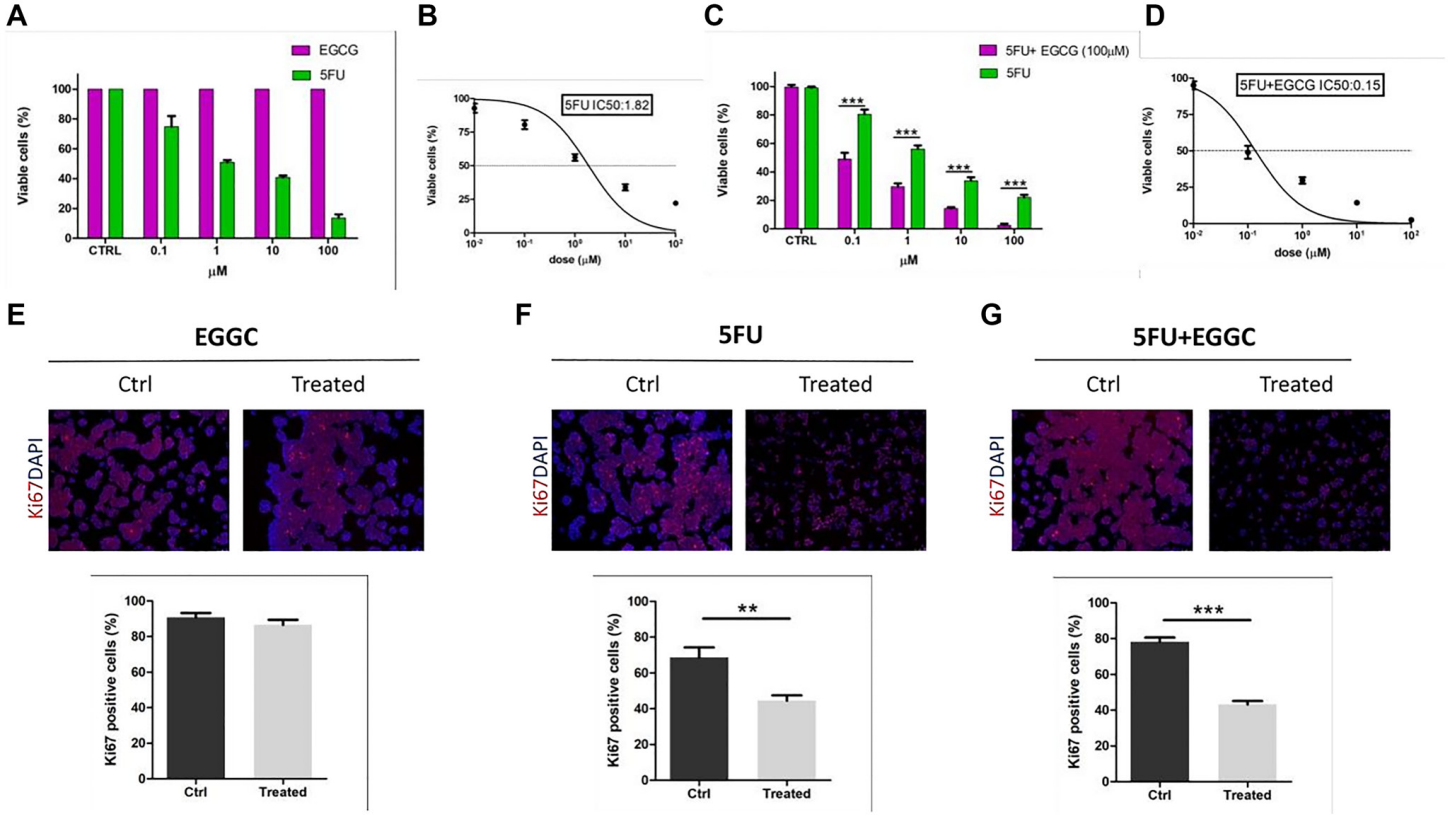
Effect of 5FU and EGCG treatments on HT29-cells. (**A**) Comparison between percentages of viable cells (by absorbance fold-change detection) after administration of 5FU and EGCG at 0.1-1-10-100 μM in HT29 cells. (**B**) Calculation of 5FU IC50 by nonlinear regression. (**C**) Comparison between percentages of viable cells after administration of 5FU alone and 5FU+EGCG (100 μM) at 0.1-1-10-100 μM in HT29 cells. (**D**) Calculation of 5FU+EGCG IC50 by nonlinear regression. (**E**) Ki67 immunofluorescences before (CTRL) and after administration of EGCG (100 μM) (scale bar = 100 μm); comparison of percentages of Ki67+ cells before (CTRL) and after treatment. (**F**) Ki67 immunofluorescences before (CTRL) and after administration of 5FU IC50 (scale bar = 100 μm); comparison of percentages of Ki67+ cells before (CTRL) and after treatment. (**G**) Ki67 immunofluorescences before (CTRL) and after administration of 5FU +EGCG IC50 (scale bar = 100 μm); comparison of percentages of Ki67+ cells before (CTRL) and after treatment (^**^ *p*-value < 0.01; ^***^ *p*-value < 0.001).

5FU, EGCG and the mixture of [5FU+EGCG] were used to investigate on their biological activities. HT29 cells were treated with the different compounds following the procedures described in the experimental section. Summarizing, cells were treated with 5FU, EGCG and [5FU+EGCG] in different concentrations and cell viability was determined. The results obtained with 5FU or EGCG concentrations in the range 0.1–100 μM are reported in Figure 1A, showing that while 5FU exhibits a clear activity, inducing the decrease of cell viability by increasing its concentration, EGCG does not express any activity, the cell viability remaining constant in all the EGCG concentration range. In other words, while 5FU response on adenocarcinoma cells maintains a dose-dependent trend, treatments with EGCG alone did not show any decrease in the percentage of cell viability. As previously shown [41, 42] the *in vitro* 5FU IC50 for HT29 cells is located in the neighborhood of 1 μM, in particular corresponding to 1.8 μM (Figure 1B).

By treating the cells with the mixtures obtained by a high EGCG concentration (100 μM, for which any direct effect on the cell viability was practically absent, see Figure 1A) and 5FU in concentrations in the range 0.1–100 μM, the results reported in Figure 1C have been obtained. In accordance with the 5FU alone response also the drug combination response on HT29 cells maintained a dose-dependent trend. Interestingly, the combined treatment resulted in a significant decrease for each concentration analyzed when compared to 5FU treatment alone (5FU vs. 5FU+EGCG: *p*-value < 0.001 at 0.1-1-10-100 μM) (Figure 1C). We calculated the drug combination IC50 for HT29 cells that resulted 0.15 μM (Figure 1D).

In order to further corroborate the different response to treatment between 5FU alone and the combination between 5FU and EGCG, Ki67 immunofluorescences analyses were performed to detect proliferation. We identified a proliferative phenotype in non-treated cells (CTRL), a situation that was completely maintained when we treated with EGCG alone at maximum concentration (100 μM) (Figure 1E). Differently, when we treated with IC50 obtained with 5FU alone and with 5FU+EGCG, cells showed a significant decrease of about 50% when compared to CTRL (5FU *p*-value = 0.0088; 5FU + EGCG *p*-value < 0.001) (Figure 1F, 1G). These results underlined the sensitivity of HT29 cells to standard chemotherapy treatment using a specific dosage but even more highlighted how the addition of EGCG to the canonical treatment can significantly contribute to cancer cell mortality.

### Behaviour of 5FU-EGCG complex in an *in vitro* human gastrointestinal digestion model

Considering the previous results related to the detection of [5FU+EGCG] complex in triple quadrupole conditions [29] and the absence of complex identification by qTOF instrument above discussed, a series of experiments were performed by direct infusion of 5FU, EGCG and the mixture of [5FU+EGCG] in the ESI source of a triple quadrupole mass spectrometer (QqQ), operating in positive and negative ion conditions [ESI(+) and in ESI(−) respectively]. Tandem mass spectrometry (MS/MS) experiments were carried out by product ion and precursor ion scans [43] of the ions of interest. These instrumental approaches lead to an enhanced specificity of the measurements, allowing to identify species present at low concentration in complex mixtures. The related instrumental parameters are reported in the experimental section.

Equimolar mixtures of [5FU+EGCG] were diluted 1:10 to a final concentration of 10^−3^ M in the different compartments (digestion juices) and diluted according to the proportions used in the aforementioned digestion protocol (Table 2). The obtained samples were further diluted 1:100 to obtain a final concentration of 10^−5^ M in 5FU and EGCG and then analyzed by direct infusions in the ESI source of the QqQ system to identify the presence of the [5FU+EGCG] complex. As shown in Table 1 the different compartments exhibit a high molecular complexity for the presence of a wide number of both organic compounds and inorganic salts. This aspect necessarily reflects on the difficulty of determination of the presence of the [5FU+EGCG] complex in the ESI spectra, in a situation analogous to that of the search of a needle in a haystack.

**Table 2:** Volumes of the digestive juices employed in the digestion model and volumes employed for the preparation of digestive fluid mixture

	*In vitro* gastrointestinal digestion model		Digestive fluid mixture (MS analysis)
	mL	Dilution factor	μL
Saliva	3	1:6, 33	300
Gastric juice	6	1:3, 16	600
Duodenal juice	6	1:3, 16	600
Bile juice	3	1:6, 33	300
Sodium carbonate solution	1	1:19	100
Final Volume	19		1900

The positive and the negative ion ESI full scan spectra of the samples related to the different compartments are reported in Tables 3 and 4. Only in few cases the presence of 5FU, EGCG and [5FU+EGCG] complex was evidenced. To achieve a better specificity, MS/MS experiments were performed and in the most of cases the presence of the complex was confirmed (Tables 3 and 4). The results obtained for the different compartments are shortly described in the following paragraphs.

**Table 3:** Ionic species identified in the ESI(+) and ESI (−) spectra of saliva, gastric and duodenal juice in presence of 5FU and EGCG and their structural identity by m/z values and product (→) and precursor (←) ion scans (MS/MS)

			Saliva	Gastric juice	Duodenal juice
**5FU**	**ESI(+)**	**Spectra**	[5FU+Na^+^] *m/z* 153 (9 × 10^7^)	[5FU+Na^+^] *m/z* 153 (5.5 × 10^7^)	[5FU+Na^+^] *m/z* 153 (6.5 × 10^7^)
		**MS/MS**			
	**ESI(−)**	**Spectra**	[5FU-H]^−^ *m/z* 129 (3.6 × 10^8^)	[5FU-H]^−^ *m/z* 129 (1.6 × 10^8^)	[5FU-H]^−^ *m/z* 129 (4.2 × 10^8^)
		**MS/MS**		[(5FU+EGCG)-H]^−^ → [5FU-H]^−^ *m/z* 587 → *m/z* 129 (2 × 10^4^)	[(5FU+EGCG)-H]^−^ → [5FU-H]^−^ *m/z* 587 → *m/z* 129 (5 × 10^4^)
**EGCG**	**ESI(+)**	**Spectra**	[EGCG+Na^+^] *m/z* 481 (1.2 × 10^8^)	[EGCG+Na^+^] *m/z* 481 (1 × 10^8^)	[EGCG+Na^+^] *m/z* 481 (5 × 10^7^)
		**MS/MS**	[EGCG+Na^+^] ← [(5FU+EGCG)+Na^+^] *m/z* 481 ← *m/z* 611 (4 × 10^6^)	[EGCG+Na^+^] ← [(5FU+EGCG)+Na^+^] *m/z* 481 ← *m/z* 611 (1 × 10^6^)	[EGCG+Na^+^] ← [(5FU+EGCG)+Na^+^] *m/z* 481 ← *m/z* 611 (3.5 × 10^5^)
	**ESI(−)**	**Spectra**	[EGCG-H]^−^ *m/z* 457 (1.7 × 10^8^)	[EGCG-H]^−^ *m/z* 457 (3 × 10^7^)	[EGCG-H]^−^ *m/z* 457 (9 × 10^7^)
		**MS/MS**	[EGCG-H]^−^ ← [(5FU+EGCG)-H]^−^ *m/z* 457 ← *m/z* 587 (2 × 10^5^)		[EGCG-H]^−^ ← [5FU+EGCG-H]^−^ *m/z* 457 ← *m/z* 587 (1 × 10^5^)
**5FU+EGCG**	**ESI(+)**	**Spectra**	[(5FU+EGCG)+Na^+^] *m/z* 611 (4 × 10^7^)	[(5FU+EGCG)+Na^+^] *m/z* 611 (3 × 10^7^)	[(5FU+EGCG)+Na^+^] *m/z* 611 (1.5 × 10^7^)
		**MS/MS**	[(5FU+EGCG)+Na^+^] → [EGCG+Na^+^] *m/z* 611 → *m/z* 481 (2.5 × 10^6^)	[(5FU+EGCG)+Na^+^] → [EGCG+Na^+^] *m/z* 611 → *m/z* 481 (3.5 × 10^5^)	[(5FU+EGCG)+Na^+^] → [EGCG+Na^+^] *m/z* 611 → *m/z* 481 (1.5 × 10^5^)
	**ESI(−)**	**Spectra**	[(5FU+EGCG)-H]^−^ *m/z* 587 (2 × 10^7^)		
		**MS/MS**	[(5FU+EGCG)-H]^−^ → [EGCG-H]^−^ *m/z* 587 → *m/z* 457 (2.5 × 10^6^)	[(5FU+EGCG)-H]^−^ → [EGCG-H]^−^ *m/z* 587 → *m/z* 457 (9 × 10^4^)	[(5FU+EGCG)-H]^−^ → [EGCG-H]^−^ *m/z* 587 → *m/z* 457 (1 × 10^6^)

**Table 4:** Ionic species identified in the ESI(+) and ESI (−) spectra of bile juice, digestive fluid mixture and digestive fluid mixture diluted 1:100 in presence of 5FU and EGCG and their structural identity by m/z values and product (→) and precursor (←) ion scans (MS/MS)

			Bile juice	Digestive fluid mixture	Digestive fluid mixture diluted 1:100
**5FU**	**ESI(+)**	**Spectra**	[5FU+Na^+^] *m/z* 153 (4 × 10^7^)		
		**MS/MS**			
	**ESI(−)**	**Spectra**	[5FU-H]^−^ *m/z* 129 (2.2 × 10^8^)	[5FU-H]^−^ *m/z* 129 (2.1 × 10^8^)	[5FU-H]^−^ *m/z* 129 (2.5 × 10^8^)
		**MS/MS**	[(5FU+EGCG)-H]^−^ → [5FU-H]^−^ *m/z* 587→ *m/z* 129 (4 × 10^4^)		
**EGCG**	**ESI(+)**	**Spectra**			[EGCG+Na^+^] *m/z* 481 (1.3 × 10^8^)
		**MS/MS**	[EGCG+Na^+^] ← [(5FU+EGCG)+Na^+^] *m/z* 481 ← *m/z* 611 (6 × 10^4^)	[EGCG+Na^+^] ← [(5FU+EGCG)+Na^+^] *m/z* 481 ← *m/z* 611 (1 × 10^3^)	[EGCG+Na^+^] ← [(5FU+EGCG)+Na^+^] *m/z* 481 ← *m/z* 611 (2 × 10^6^)
	**ESI(−)**	**Spectra**	[EGCG-H]^−^ *m/z* 457 (7 × 10^7^)		[EGCG-H]^−^ *m/z* 457 (4 × 10^7^)
		**MS/MS**	[EGCG-H]^−^ ← [(5FU+EGCG)-H]^−^ *m/z* 457 ← *m/z* 587 (2 × 10^5^)		[EGCG-H]^−^ ← [5FU+EGCG-H]^−^ *m/z* 457 ← *m/z* 587 (8 × 10^6^)
**5FU+EGCG**	**ESI(+)**	**Spectra**			[(5FU+EGCG)+Na^+^] *m/z* 611 (2.5 × 10^7^)
		**MS/MS**	[(5FU+EGCG)+Na^+^] → [EGCG+Na^+^] *m/z* 611 → *m/z* 481 (1.6 × 10^5^)	[(5FU+EGCG)+Na^+^] → [5FU+Na^+^] *m/z* 611 → *m/z* 153 (5 × 10^4^)	[(5FU+EGCG)+Na^+^] → [EGCG+Na^+^] *m/z* 611 → *m/z* 481 (1.4 × 10^6^)
	**ESI(−)**	**Spectra**			[(5FU+EGCG)-H]^−^ *m/z* 587 (2 × 10^7^)
		**MS/MS**	[(5FU+EGCG)-H]^−^ → [EGCG-H]^−^ *m/z* 587 → *m/z* 457 (2 × 10^5^)		[(5FU+EGCG)-H]^−^ → [EGCG-H]^−^ *m/z* 587 → *m/z* 457 (6 × 10^6^)

### Saliva solution

The first experiments were performed by direct introduction of diluted mixtures of 5FU and EGCG in the saliva juice. The results obtained by full scan ESI(+) are reported in the first column of Table 3, showing the favoured formation of adducts with Na^+^ of either 5FU and EGCG (at *m/z* 153 and 481 respectively). Interestingly also the signal of the ion related to the complex [(5FU+EGCG)+Na^+^] is detected at *m/z* 611 with a high intensity (4 × 10^7^ DAC units). Product ion scan (MS/MS in Table 3) performed on [(5FU+EGCG)+Na^+^] ion shows the formation of EGCG sodium adduct only, indicating an higher affinity *versus* Na^+^ of this molecules with respect to that of 5FU. The presence of the complex [(5FU+EGCG)+Na^+^] has been confirmed by precursor ion scan performed on ion [EGCG+Na^+^] (Table 3). In principle, the formation of [(5FU+EGCG)+Na^+^] could be considered to originate by the presence of abundant Na^+^ ions present in the biological substrate. In other words Na^+^ ions could be responsible for the complex formation, i.e., it could be the catalyzer for the [(5FU+EGCG)+Na^+^] formation, following the reaction pathways:


$$\begin{array}{c} {\text{[EGCG+Na}}^{+}\text{] + 5FU }\to {\text{[(5FU+EGCG)+Na}}^{+}\text{]}\\ {\text{EGCG+[5FU+Na}}^{+}\text{] }\to \;{\text{[(5FU+EGCG)+Na}}^{+}\text{]} \end{array}$$

To investigate on this hypothesis, a series of experiments in negative ion ESI conditions were performed. In these conditions deprotonated species are observed at *m/z* 129, 457 and 587 (corresponding to [5FU-H]^−^, [EGCG-H]^−^ and [(5FU+EGCG)-H]^−^, see Table 3). The last ion can be considered a clear evidence of the presence of the complex in the injected solution, which in positive ion ESI conditions is directly cationized by Na^+^. Product ion scan performed on deprotonated complex show the formation of both deprotonated 5FU and EGCG (Table 3). Furthermore, complex [(5FU+EGCG)-H]^−^ has been detected by precursor ion scan performed on deprotonated [EGCG-H]^−^ (Table 3).

### Gastric juice

As shown in the second column of Table 3, the bimolecular complex is detected in the ESI(+) spectrum as sodium adduct [(5FU+EGCG)+Na^+^]. Its chemical nature has been confirmed by both MS/MS data performed on [(5FU+EGCG)+Na^+^] (product ion scan), leading to the formation of [EGCG+Na^+^], and precursor ion scan performed on protonated [EGCG+Na^+^] (Table 3).

In negative ion ESI conditions (Table 3) deprotonated species of 5FU and EGCG are observed, but the bimolecular complex is practically undetectable in the ESI spectrum. However, its presence has been confirmed by product ion spectrum performed on [(5FU+EGCG)-H]^−^, leading to [EGCG-H]^−^ species.

### Duodenal juice

In this case the complex is detectable (even if in low abundance) in the ESI(+) spectrum (3rd column of Table 3) and its presence has been confirmed by both MS/MS data performed on [(5FU+EGCG)+Na^+^] and precursor ion scan performed on [EGCG+Na^+^] species (see Table 3).

In negative ion condition (Table 3) deprotonated species are observed, even if the signal related to bimolecular complex is of very low intensity. However, its presence has been confirmed by both product ion scan performed on [(5FU+EGCG)-H]^−^ and precursor ion scan performed on deprotonated [EGCG-H]^−^ (Table 3).

### Bile juice

In the ESI(+) spectrum obtained for this digestive juice the complex is undetectable (see first column of Table 4) but its presence has been confirmed by both MS/MS data performed by product ion scan of [(5FU+EGCG)+Na^+^] species and precursor ion scan performed on protonated [EGCG+Na^+^].

In negative ion condition deprotonated species of 5FU and EGCG are observed, even if the bimolecular complex is practically undetectable. The presence of complex [5FU+EGCG] has been confirmed by both MS/MS data performed on [(5FU+EGCG)-H]^−^ and deprotonated [EGCG-H]^−^ (Table 4).

### Sodium carbonate solution

In this case, due to the high abundance of Na^+^ ions, the complex is detectable only in positive ion conditions. Its presence has been confirmed by both product and precursor ion scan data. In negative ion conditions the only ion detectable is due to [5FU-H]^−^.

### Digestive fluid mixture

Before to undertake experiments by the *in vitro* gastrointestinal digestion model of 5FU and EGCG, it was considered of interest to study the behaviour of these compounds in the sample obtained by mixing the different compartments employed for the digestion process. The added mixture of 5FU and EGCG was diluted 1:10 to achieve a final concentration of 10^−3^ M in the digestive fluid mixture, prepared accordingly to the proportions used in Table 2. The samples thus obtained were diluted 1:100 to obtain a final concentration of 10^−5^ M in 5FU and EGCG and then analyzed by direct infusions in the ESI source of the QqQ system, to identify the possible presence of the [5FU+EGCG] complex. Considering the high complexity of the different compartments of the digestion model, it can be easily recognized the higher complexity of the digestive fluid mixture and how it would reflect on the difficulty to identify the compounds of interest.

As matter of fact it was not possible to highlight the presence of 5FU, EGCG and bimolecular complex both in positive and negative ion conditions (see 2nd column of Table 4). This result can be reasonably ascribed to the presence of highly concentrated organic molecules and inorganic salts, leading to “ion suppression” phenomena, often observed in ESI conditions. This hypothesis is confirmed by diluting 1:100 the concentration of digestive fluid mixture before adding 5FU and EGCG: in these conditions by MS the complex is easily detectable (see 3rd column of Table 4) and its chemical nature is confirmed by product and precursor ion scans performed on the species of interest.

The data reported and summarized in Tables 3 and 4, suggest that 5FU, EGCG and their bimolecular complex seem to survive to the quite severe chemical conditions related to the *in vitro* digestion. Consequently the *in vitro* gastrointestinal model, described in the experimental section, has been employed to study the behaviour of 5FU and EGCG mixtures.

### Experiments by *in vitro* gastrointestinal digestion model

The digestion procedure employed in the present investigation was that proposed by Walczak et al. [44], described in detail in the experimental section. When the digestion protocol is applied to 5FU and EGCG (in a concentation of 10^−2^ M), the final supernatant obtained after the digestive procedure was analyzed, after 1:100 dilution, by direct infusion into the ESI source, but in this case was not possible to evidence the presence of 5FU, EGCG and the related bimolecular complex also by using the MS/MS methods that in the above-described conditions (see data reported in Tables 3 and 4) allowed to obtain valid results. In order to possibly gain further specificity the supernatant sample was treated by Zip-Tip to reduce the salt concentration. Also in these conditions, there was no evidence for the analyte presence after infusion in the QqQ system. Considering that the final 5FU and EGCG concentrations after digestion were in the order of 10^−6^ M, a further attemp was devoted to the employement of an analyte sample concentration procedure, based on the treatment of the supernatant sample by Select HLB C18 column (see experimental). Again, also this approach did not lead to any significative results.

## DISCUSSION

5FU is an extensively used drug in CRC adjuvant chemotherapy and still represents the backbone of different multimodal treatments [45]. Recently it has been reported [22] that Epigallocatechin Gallate (EGCG) enhances the sensitivity of colorectal cancer cells to 5FU by Inhibiting GRP78/NF-κB/miR-155- 5p/MDR1 pathway. Consequently, EGCG can be considered as a potential antitumor drug. Yang et al. revealed that the combination of EGCG enhanced the chemosensitivity of HCC cells to 5-FU by inhibiting COX-2 expression and PGE2 secretion [18–21]. Hu et al. [46] suggested that EGCG minimized the drug resistance of cisplatin and oxaliplatin in colorectal cancer cells through activating autophagy.

At present, the resistance to chemotherapy and its toxic side effects are the main barriers in cancer therapy. Therefore, exploring the mechanism of EGCG enhancing tumor chemotherapy sensitivity would have a profound impact on its clinical application.

Looking at these findings and considering our previous experience on the formation of bimolecular non-covalent complexes described in the introduction section, in particular on the formation of [5FU+EGCG] complex [29], it was considered of interest to undertake the present investigation on its possible role in the biological activity described for 5FU and EGCG mixtures.

The possible interaction between 5FU and catechins can be considered as due to the high electron density present on the fluorine atom in 5FU and to the low electron density on the hydrogen atoms of the hydroxyl groups in catechins. The easy formation of hydrogen bonding activated by 5FU, with the formation of non-covalent complexes, has been recently discussed by Deepa et al. [47] in the case of base pairs. The nature of the hydrogen bond so formed was analysed through different theories, proving that 5FU binds strongly with usual and mismatch base pairs. These results suggest that the hydroxybenzene substituent of catechins can be considered responsible for the complex formation with 5FU.

The first step of the present investigation was the evaluation of the instrumental approach employed (LC-qTOF, see experimental) in the characterization of compounds 5FU, EGCG and the related bimolecular complex. The total ion chromatogram obtained by injection of 1 ng/μL solutions of 5FU and EGCG still show the presence of 5FU and EGCG molecular species, but any signal related to the complex was undetectable. This result can be explained by the low yield of the formation of the complex, as well as by its thermal instability, due to the operative conditions of the qTOF instrument. In fact, an ion source temperature of 120°C and a desolvation gas (sheath gas) temperature of 600°C are present and the latter could be responsible for the decomposition of the bimolecular, non-covalent complex. Hence the MS data seems, at first sight, to exclude the formation of the complex, even if previous results obtained by triple quadrupole (QqQ) instrument operating in softer conditions unequivocally proved its formation [29]. Then the complex has to be considered in principle present in the solution of [5FU+EGCG].

As reported in the introduction section, many researches have been devoted to the activity of [5FU+EGCG], showing that EGCG enhances the sensitivity of cancer cells to 5FU. Then the response of human colon adenocarcinoma cell line HT29 with respect to 5FU, EGCG and [5FU+EGCG] was tested. In comparison with other adenocarcinoma cell lines, HT-29 is not the most sensitive cell line to EGCG treatments with respect to the growth inhibition and the induction of cell death [48]. The reason for this observation may partially be attributed to the fact that EGCG is extensively metabolized to methylated and glucuronidated conjugates in HT29 cells, and then actively pumped out of cells by multi-drug resistance-related proteins (MRPs) as shown in recent study about the uptake, biotransformation, and efflux of EGCG in HT-29 cells [49].

HT29 cells were treated with different concentrations of 5-FU alone, EGCG alone for 72 hours. Analogously the cells were treated with the [5FU+EGCG] mixture (with a constant EGCG 100 μM concentration and 5FU concentrations varying from 0.1 μM to 100 μM). After 72 h post-treatment 100 μL of medium were drawn, filtered and centrifuged (see Experimental). The samples so obtained were firstly analyzed by mass spectrometry: 5 μL were injected in the LC/qTOF instrument, leading to a complex total ion chromatogram due to the high number of molecular species present in the medium, which reasonably could induce possible ion suppression phenomena. As matter of fact by reconstructed ion chromatograms 5FU is detectable, while neither EGCG nor the [5FU+EGCG] complex are detectable. It is interesting to observe that in the reconstructed ion chromatogram (RIC) of 5FU different peaks, isobaric with deprotonated 5FU have been found: they can be due to the presence of tautomeric forms, as those described by Wielinska et al. [50].

The results obtained in the study of cell viability with 5FU and EGCG concentrations in the range 0.1–100 μM are reported in Figure 1A, showing that 5FU exhibits a clear activity, with the decrease of cell viability by increasing the 5FU concentration. On the contrary EGCG alone, in the concentration range of 0.1–100 μM does not express any activity, the cell viability remaining practically constant in all the concentration range. This last result is at first sight different to that discussed by La et al. [22], showing a decrease of cell viability by increasing the EGCG concentration. However, it must be stressed that in the research of La et al. the EGCG concentration effect was studied in the range 0–700 μM. In the present study it was choosen to study the effect of low concentrations of either EGCG and, over all, 5FU in order to reduce possibly the toxic effects of the therapy.

By treating the cells with the mixtures obtained by a higher EGCG concentration (100 μM, for which any direct effect on the cell viability was practically absent (see Figure 1A) and a 5FU concentrations in the range 0.1–100 μM, the results reported in Figure 1B have been obtained. In this case a strong effect of EGCG becomes present. Interestingly, it was observed that the combined treatment resulted in a significant decrease for each concentration analyzed when compared to 5FU treatment alone (5FU vs. 5FU+EGCG: *p*-value < 0.001 at 0.1-1-10-100 μM) (Figure 1C). We calculated the drug combination IC50 for HT29 cells that resulted 0.15 μM (Figure 1D). In comparison to 0.1 μM 5-FU alone, the cotreatment with 100 μM EGCG caused additional reduction to 30% in cell viability. In addition, drug sensitization fold refers to the ability of a certain sensitizer to maximize damage of antidrugs on tumor cells. The calculated results showed that when cotreated with 100 μM EGCG, the sensitivity of HT29 cells to 5-FU was increased by 12- fold. These data are in agreement with those given by other authors [20–22], proving the effects of EGCG in the enhancement of 5FU activity.

A question remains open: are the obtained results due to concurrent actions of 5FU and EGCG, promoting different pathways activating the 5FU activity, or is a new molecular entity, i.e., the non-covalent complex [5FU+EGCG], responsible for the observed behaviour? In the present investigation any trace of complex has not been evidenced, on the contrary of what observed in a previous investigation performed by a QqQ instrument [29]. This result can be due to the instrumental aspects related to the LC/ESI/qTOF approach herein employed: this choice was done to gain analytical specificity by its high resolution conditions, but, as above discussed, the operative conditions of the ion source could be lesive for the complex survival.

As described in the introduction section, orally administered therapeutic agents must exhibit gastrointestinal stability and undergo efficient uptake. Consequently, to verify the possible therapeutic activity of the 5FU, EGCG and of the 5FU-EGCG complex, their survival in the gastro-intestinal region must be evaluated. For these reasons an investigation on their behaviour in the different compartments (saliva, gastric, duodenal and bile juices, whose composition is reported in Table 1) has been considered. For this aim equimolecular solutions of 5FU and EGCG were added to the different digestive juices in the conditions described in the experimental section. Considering the results previously obtained [29] and the failure of qTOF for the 5FU-EGCG complex identification, for this investigation the QqQ approach was employed and, to gain specificity, tandem mass spectrometry experiments were systemically performed. The results so obtained and summarized in Tables 3 and 4, prove that 5FU, EGCG and their bimolecular complex survive to the quite severe chemical conditions related to the *in vitro* treatment with digestive juices. However, in the digestive fluid sample obtained by mixing the digestion fluids described in Table 1 in the ratio reported in Table 2, the signals obtained by MS methods of EGCG, as well as of 5FU and 5FU-EGCG complex are completely absent. This may be due to an ineffectiveness of the analytical method employed for a so complex mixture. It must be considered that in the analysis in ESI conditions complex mixtures can lead to ion suppression effect, a phenomenon where coeluting species suppress the signal(s) of the analyte(s): with high concentrations of interfering compounds, the signal of analyte becomes severely suppressed and, consequently, undetectable. To verify this aspect the solution of the digestive juice mixture was diluted 1:100 to reduce the concentration of the organic species and inorganic salts present in the injected sample. This treatment surely strongly reduces the concurrency of matrix molecules in the ionization step as well as their interactions with the analyte molecules. Both the positive and negative ion ESI spectra so obtained show the presence of ions due to 5FU, EGCG and, more important, [5FU+EGCG] complex, whose identity was confirmed by MS/MS experiments. Then the obtained results seem to confirm the survival of the complex after treatment with the digestive juice mixture.

Then the digestion protocol proposed by Walczak et al. [44] was applied to 5FU and EGCG (in a concentration of 10^−2^ M). The supernatant obtained after the digestive procedure was analyzed, after 1:100 dilution, by direct infusion into the ESI source. In this case was not possible to evidence the presence of 5FU, EGCG and the related bimolecular complex, also by using the MS/MS methods that in the above described conditions allowed to obtain the identification of 5FU, EGCG and [5FU+EGCG] complex. In order to possibly gain further specificity the supernatant sample was treated by Zip-Tip to reduce the salt concentration. Also the sample so obtained, after infusion in the QqQ system, did not give evidence for the presence of the analytes of interest. Considering that the final 5FU and EGCG concentrations were, after digestion treatment, in the order of 10^−4^ M, a further attemp was devoted to the employement of an analyte sample concentration procedure, based on the treatment of the supernatant sample by Select HLB C18 column (see experimental). Also this approach did not lead to any significative results.

These results seem to be contradictory to those obtained by single or mixed digestion juices above described. The negative results obtained by applying the digestive protocol could be explained by different phenomena. It is relevant to underline that in the supernatant sample neither signals related to the bimolecular complex nor those due to to intact 5FU and EGCG are obtained, suggesting that they do not survive to, or become undetectable after, the digestion protocol treatment. This behaviour might be ascribed, in the case of 5FU to its low solubility in water (1 mg/mL) which would lead to the formation of a precipitate. In the case of EGCG, its disappearance could be due to its chemical unstability. In fact the data obtained by Krook and Hagerman [51] showed that polyphenol in digestion experiments can exibit different fates and effects, depending on whether they are injected as compounds, as constituents of beverages, or as constituents of foods. In particular it has been shown [35, 36] that EGCG undergoes oxidation and rearrangement reactions at neutral to basic pH and in the presence of dissolved oxygen. The stability of EGCG in the mammalian digestive system was evaluated using solutions at pH 1.8, similar to the stomach, and pH 7.0, similar to the duodenum under reduced oxygen concentration [52]. EGCG was stable at pH 1.8 but in the absence of food or digestive components 90% of EGCG was lost. These two factors can exhibit a role, but another point must be considered, related to the analyte concentrations present in the supernatant samples (10^−4^ M) one order of magnitude lower than that present for the experiments performed on single digestion juice and on their mixture (10^−3^ M). A further aspect must be taken in account: reasonably the procedure proposed by Walczak et al. [44] was too extreme. Two hours in acidic conditions to mimic digestion seem to be too long for maintain unaltered the non-covalent molecular complex. To verify this aspect further investigation are required to identify and evaluate new suitable conditions to simulate the complex movement through the gastrointestinal tract.

## MATERIALS AND METHODS

### Samples

5FU (99% purity), EGCG (95% purity) and DMSO (99% purity) were purchased by Sigma Aldrich (Sigma Aldrich, S.Louis, MO, USA). Filtered, deionized water was purified using a Milli-Q Academic/Quantum EX system (Millipore, Milford, MA, USA). Absolute MeOH LC-MS grade (cod. H353) and formic acid LC-MS grade (cod. H411) were purchased from ROMIL (ROMIL Ltd, Cambridge, GB). The artificial digestive juices for *in vitro* digestion were a kind gift from Ecam Ricert, prepared according to literature [39].

### Roles of 5 fluorouracil and epigallocatechin-3-gallate on HT-29 cell death

In order to verify the instrumental response by LC/MS on the compounds of interest, solutions at different concentrations of the two analytes in H_2_O/MeOH were prepared: (i) 5FU solutions: 1ng/μL (7.7 μM); 10 ng/μL (76.9 μM). (ii) EGCG solutions: 1 ng/μL (2.2 μM), 10 ng/μL (21.8 μM). The solutions of the two compounds were employed to produce mixed solutions with 5FU/EGCG molar ratios 3.5 and 0.35. All these solutions were analyzed by LC/MS by the qTOF instrument.

For investigating on the biological behaviour of the two compounds, solutions different from those above described were prepared. In this case both 5FU and EGCG were solubilized in DMSO both at a 100 mM concentration. These solutions were stored at 4°C and from them different 10 μL aliquots were drawn. After dilution in the cell medium to 1000 μM concentration, different concentrations were obtained by dilution with the cell medium: 100 μM, 10 μM, 1 μM and 0.1 μM. These solutions were directly deposited into the well plate.

After 72 h post-treatment 100 μL of medium were drawn, filtered by Amicon Ultra 0.5 mL centrifugal filters, purchased by Sigma Aldrich (Sigma Aldrich, S.Louis, MO, USA),and centrifuged.

All these solutions were analyzed by LC/MS by the qTOF instrument.

### Simulation of the behavior of [5FU+EGCG] in different compartments of the gastro intestinal model by mass spectrometry

5FU and EGCG were dissolved in methanol at a concentration of 5 mg/mL (10^−2^ M).

Mixtures of 5FU and catechins were prepared in 1:1 molar ratio at a final concentration of 10^−2^ M and stored overnight at room temperature to promote interactions at the molecular level.

To simulate the behavior of [5FU+EGCG] in different compartments of the gastro intestinal model saliva, gastric juice, duodenal juice, bile juice and sodium carbonate solution (Table 1) were employed.

Equimolar mixtures of [5FU+EGCG] were diluted 1:10 to a final concentration of 10^−3^ M in the different compartments, diluted according to the proportions used in the Ecam Ricert digestion protocol (reported in Table 2). The samples thus obtained were diluted 1:100 to a final concentration of 10^−5^ M and then analyzed by direct infusions in the QqQ System to identify the presence of the [5FU+EGCG] complex. The mixtures [5FU+EGCG] were introduced into the digestive juicy mixture and further measurements were carried out by means of QqQ (see Table 2). The digestion procedure was that proposed by Walczak et al. [44], which can be summarized as follows: 452 μL of the 10^−2^ M solution of [5FU+EGCG] were added to 3 mL of saliva. The mixture was incubated for 5 min at 37°C, kept under constant agitation at 100 rpm. Subsequently 6 mL of gastric juice was added to the mixture, the sample was further incubated under agitation at 37°C for 2 hours. Subsequently 6 mL of duodenal, 3 mL of bile juice and 1 mL of sodium bicarbonate solution were added and kept in agitation for 2 hours. At the end of the *in vitro* digestion process, the digestion tubes are centrifuged for 5 min at 2750 g, yielding the supernatant (in which there should be the compounds of interest) and the digested matrix (the pellet). After this treatment the mixture of [5FU+EGCG] concentration decreases in supernatant to 10^−4^ M.

The supernatant obtained after the *in vitro* digestive procedure was analyzed, after 1:100 dilution (so obtaining a 10^−6^ M concentration of [5FU+EGCG]), by direct infusion into the ESI source but in this case was not possible to evidence the presence of 5FU, EGCG and the related bimolecular complex also by using the MS/MS methods that in the above-described conditions allowed to obtain valid results.

In order to possibly gain further specificity the supernatant sample was treated by Millipore ZipTip_C18_ purchased by Sigma Aldrich (Sigma Aldrich, S.Louis, MO, USA) in order to reduce the salt concentration. Also the sample so obtained, after infusion in the QqQ system, did not give evidence for the analyte presence. ZipTips are 10 μL pipette tips containing C18 used for desalting and concentrating peptides or small proteins.

Considering that the final 5FU and EGCG concentration were, after digestion treatment in the order of 10^−4^ M, a further attemp was devoted to the employement of a analyte sample concentration procedure, based on the treatment of the supernatant sample by SPE (solid phase extraction) by Supel-Select HLB C18 column, volume 1 mL purchased by Sigma Aldrich (Sigma Aldrich, S.Louis, MO, USA), leading to a concentration 10^−3^ M of [5FU+EGCG]. Also, this approach did not lead to any significative results.

### Mass spectrometric measurements

#### LC/MS measurements by qTOF

An ACQUITY UPLC H-Class system coupled to a Waters Xevo G2-XS QTOF mass spectrometer (MS) (Waters UK, Elstree, UK) was used. All chromatographic and MS equipments were purchased from Waters Corporation (Milford, MA, USA). Chromatographic separations were achieved using a Synergi Fusion C18, 4 μm, 2 × 50 mm (Phenomenex, Torrance, California, USA) column. Analytical column chromatography was performed at 40°C. The mobile phase consisted of ultrapure H_2_O/0.1% HCOOH (A) and MeOH/0.1% HCOOH (B) according to the following elution gradient: initial at 95% solvent A for 0.5 min, then decreased to 55% in 24.5 min, then further decreased to 2% in 1.0 min and kept for 4.0 min; it returned to initial conditions in 1.0 min and maintained for 4.0 min for reconditioning. The flow rate was set at 0.25 mL/min. MS experiments were performed using a Waters Xevo G2-XS QTOF mass spectrometer connected to the ACQUITY UPLC H-Class system via Z Spray dualorthogonalspray source. ESI ionization was performed in negative ion mode and sensitivity analyser modes for quadrupole time of flight (qTOF)-MS data acquisition. The mass spectrometer parameters were set as follows: mass range, *m/z* 50–2000; capillary voltage, 2.0 kV; sampling cone, 20 V; source offset, 80 V; source temperature, 120°C; desolvation gas (sheath gas) temperature, 600°C; cone gas, 50 L/h; desolvation gas, 1000 L/h. Analyses were performed in full scan mode, and the scan time was set to 0.2 s. To ensure for mass accuracy and reproducibility of the optimized MS conditions, leucine enkephalin (m/z 554.2615 in negative mode) was used as a reference (lock mass) at a concentration of 100 pg/μLand a flow rate of 10 μL/min. The reference was injected into the MS instrument every 10 s. The instrument was calibrated using sodium iodide (NaI) solution as the calibration standard to achieve mass accuracies of <0.5 mDa.

#### Mass spectrometric measurements by triple quadrupole (QqQ)

Mass spectrometry measurements on the 5FU-EGCG mixtures were performed by using an API 4000 triple quadrupole mass spectrometer (AB SCIEX, MA, USA).

The mixtures 5FU-EGCG solutions were infused by the use of a programmable syringe pump (KD Scientific, MA, USA), at a flow rate of 300 μL/h. ESI source parameters were as follows: source temperature, 100°C; curtain gas (nitrogen), 15 psi; nebulizer gas (air) GS1 and GS2, 10 and 0 psi, respectively.

- *Full Scan Spectra*. Full scan spectra in the positive ion mode were recorded with ion spray voltage set at +4500 V, entrance potential at 10 V, and declustering potential at 20 V; for negative ion measurements ion spray voltage was set at −4500 V, entrance potential at −10 V, and declustering potential at −20 V.
- *MS/MS Spectra* (Precursor Ion and Neutral Loss Scans). For collisional experiments CAD was set at 4 (arbitrary units); for positive ion measurements collision cell exit potential (CXP) and collision energy (CE) were respectively 15 and 30 V, while for negative measurements CXP and CE were −15 and −30 V.

### Cells maintenance and expansion

The human colon adenocarcinoma cell line HT-29 was obtained from American Type Culture Collection (ATCC^®^ HTB-38™) and maintained in RPMI-1640 medium supplemented with 10% fetal bovine serum, 1% L-Glutamine and 1% pen/strep antibiotic solution (growth medium) in humidified atmosphere at 37°C in 5% CO2. Cells were routinely tested for Mycoplasma through MycoAlert^®^ Kit (Promega). HT-29 derived from a primary tumor of a 44-year-old caucasian female, using the explant culture method. HT-29 show epithelial behavior *in vitro* forming a tight monolayer, while exhibiting similarity to enterocytes from the small intestine. They have tumorigenic potential and are positive for expression of c-myc, K-ras, H-ras, N-ras, Myb, sis and fos oncogenes. The p53 antigen is overproduced, and there is a G/A mutation in codon 273 of the p53 gene resulting in an Arg/His substitution.

### Immunofluorescence analysis

For immunofluorescence analysis 5 × 10^3^ cells were seeded in a 96-well plate and treated for 72 hours. Once the time point was reached, cells were washed twice in 1X PBS and fixed in 4% paraformaldehyde (PFA) (Sigma Aldrich) for 10 minutes at 4°C and then rinsed twice in 1X PBS. Permeabilization was obtained with 10 minutes at RT in 0.5% Triton X-100 (Fluxa) in 100 μL of 1X PBS. Cells were washed in 1X PBS and then incubated with 50 μL of primary antibody diluted in 1% Bovine Serum Albumin (BSA) (Sigma Aldrich). Rabbit anti-human Ki67 (Abcam) primary antibody was used to test the presence of proliferative cells. Then cells were washed twice in 1X PBS for 5 minutes each and were then incubated for 45 minutes at RT in the dark with secondary antibodies (Alexa Fluor chicken anti-rabbit 594, diluted in 1% BSA. After double washing with 1X PBS, nuclei were stained with 50 μL of 1:10000 HOECHST (Life Technologies) for 15 minutes at RT in the dark. A last wash was performed in 1X PBS and cells were maintained in 1X PBS to be analysed. Images were collected using a fluorescence inverted microscope (Leica B5000). The number of Ki67+ cells divided by the number of total nuclei, was calculated to quantify the number of proliferating cells. The number of proliferating cells was acquired using ImageJ software.

### Drug treatment and cytotoxicity assay

HT29 were seeded at 10 × 10^3^ cells per well in 96-well plates and treated with different concentrations of 5FU alone (from 0.1 μM to 100 μM), EGCG alone (from 0.1 μM to 100 μM) for 72 hours. Analogously the cells were treated with the [5FU+EGCG] mixture (with a constant EGCG 100 μM concentration and 5FU from 0.1 μM to 100 μM).

Cell viability was determined 72 h post-treatment by reading the absorbance using Tecan Microplate Reader Spark^®^. The treatment response for each culture setting was standardized to the corresponding untreated cultures. Similarly, the Resazurin Reagent (Abcam) was used for the Inhibitory Concentration 50% (IC_50_) determination using GraphPad Prism software 6.

## References

[1] Bray F , Ferlay J , Soerjomataram I , Siegel RL , Torre LA , Jemal A . Global cancer statistics 2018: GLOBOCAN estimates of incidence and mortality worldwide for 36 cancers in 185 countries. CA Cancer J Clin. 2018; 68:394–424. 10.3322/caac.21492. 30207593

[2] Longley DB , Harkin DP , Johnston PG . 5-fluorouracil: mechanisms of action and clinical strategies. Nat Rev Cancer. 2003; 3:330–38. 10.1038/nrc1074. 12724731

[3] Johnston PG , Kaye S . Capecitabine: a novel agent for the treatment of solid tumors. Anticancer Drugs. 2001; 12:639–46. 10.1097/00001813-200109000-00001. 11604550

[4] Giacchetti S , Perpoint B , Zidani R , Le Bail N , Faggiuolo R , Focan C , Chollet P , Llory JF , Letourneau Y , Coudert B , Bertheaut-Cvitkovic F , Larregain-Fournier D , Le Rol A , et al. Phase III multicenter randomized trial of oxaliplatin added to chronomodulated fluorouracil-leucovorin as first-line treatment of metastatic colorectal cancer. J Clin Oncol. 2000; 18:136–47. 10.1200/JCO.2000.18.1.136. 10623704

[5] Douillard JY , Cunningham D , Roth AD , Navarro M , James RD , Karasek P , Jandik P , Iveson T , Carmichael J , Alakl M , Gruia G , Awad L , Rougier P . Irinotecan combined with fluorouracil compared with fluorouracil alone as first-line treatment for metastatic colorectal cancer: a multicentre randomised trial. Lancet. 2000; 355:1041–47. 10.1016/s0140-6736(00)02034-1. 10744089

[6] Longley DB , Johnston PG . Molecular mechanisms of drug resistance. J Pathol. 2005; 205:275–92. 10.1002/path.1706. 15641020

[7] Chen PN , Chu SC , Kuo WH , Chou MY , Lin JK , Hsieh YS . Epigallocatechin-3 gallate inhibits invasion, epithelial-mesenchymal transition, and tumor growth in oral cancer cells. J Agric Food Chem. 2011; 59:3836–44. 10.1021/jf1049408. 21388137

[8] Hwang YS , Park KK , Chung WY . Epigallocatechin-3 gallate inhibits cancer invasion by repressing functional invadopodia formation in oral squamous cell carcinoma. Eur J Pharmacol. 2013; 715:286–95. 10.1016/j.ejphar.2013.05.008. 23707351

[9] Jee HG , Lee KE , Kim JB , Shin HK , Youn YK . Sulforaphane inhibits oral carcinoma cell migration and invasion *in vitro* . Phytother Res. 2011; 25:1623–28. 10.1002/ptr.3397. 21413088

[10] Pons-Fuster López E , Wang QT , Wei W , López Jornet P . Potential chemotherapeutic effects of diosgenin, zoledronic acid and epigallocatechin-3-gallate on PE/CA-PJ15 oral squamous cancer cell line. Arch Oral Biol. 2017; 82:141–46. 10.1016/j.archoralbio.2017.05.023. 28641180

[11] Wang ST , Cui WQ , Pan D , Jiang M , Chang B , Sang LX . Tea polyphenols and their chemopreventive and therapeutic effects on colorectal cancer. World J Gastroenterol. 2020; 26:562–97. 10.3748/wjg.v26.i6.562. 32103869 PMC7029350

[12] Tedeschi E , Menegazzi M , Yao Y , Suzuki H , Förstermann U , Kleinert H . Green tea inhibits human inducible nitric-oxide synthase expression by down-regulating signal transducer and activator of transcription-1alpha activation. Mol Pharmacol. 2004; 65:111–20. 10.1124/mol.65.1.111. 14722242

[13] Shin JH , Jeon HJ , Park J , Chang MS . Epigallocatechin-3-gallate prevents oxidative stress-induced cellular senescence in human mesenchymal stem cells via Nrf2. Int J Mol Med. 2016; 38:1075–82. 10.3892/ijmm.2016.2694. 27498709 PMC5029951

[14] Lee JH , Jin H , Shim HE , Kim HN , Ha H , Lee ZH . Epigallocatechin-3-gallate inhibits osteoclastogenesis by down-regulating c-Fos expression and suppressing the nuclear factor-kappaB signal. Mol Pharmacol. 2010; 77:17–25. 10.1124/mol.109.057877. 19828731

[15] Vali B , Rao LG , El-Sohemy A . Epigallocatechin-3-gallate increases the formation of mineralized bone nodules by human osteoblast-like cells. J Nutr Biochem. 2007; 18:341–47. 10.1016/j.jnutbio.2006.06.005. 16963251

[16] Ho YC , Yang SF , Peng CY , Chou MY , Chang YC . Epigallocatechin-3-gallate inhibits the invasion of human oral cancer cells and decreases the productions of matrix metalloproteinases and urokinase-plasminogen activator. J Oral Pathol Med. 2007; 36:588–93. 10.1111/j.1600-0714.2007.00588.x. 17944751

[17] Chen PS , Shih YW , Huang HC , Cheng HW . Diosgenin, a steroidal saponin, inhibits migration and invasion of human prostate cancer PC-3 cells by reducing matrix metalloproteinases expression. PLoS One. 2011; 6:e20164. 10.1371/journal.pone.0020164. 21629786 PMC3100339

[18] Yang XW , Wang XL , Cao LQ , Jiang XF , Peng HP , Lin SM , Xue P , Chen D . Green tea polyphenol epigallocatechin-3-gallate enhances 5-fluorouracil-induced cell growth inhibition of hepatocellular carcinoma cells. Hepatol Res. 2012; 42:494–501. 10.1111/j.1872-034X.2011.00947.x. 22221825

[19] Lee JC , Chung LC , Chen YJ , Feng TH , Chen WT , Juang HH . Upregulation of B-cell translocation gene 2 by epigallocatechin-3-gallate via p38 and ERK signaling blocks cell proliferation in human oral squamous cell carcinoma cells. Cancer Lett. 2015; 360:310–18. 10.1016/j.canlet.2015.02.034. 25721086

[20] Toden S , Tran HM , Tovar-Camargo OA , Okugawa Y , Goel A . Epigallocatechin-3-gallate targets cancer stem-like cells and enhances 5-fluorouracil chemosensitivity in colorectal cancer. Oncotarget. 2016; 7:16158–71. 10.18632/oncotarget.7567. 26930714 PMC4941304

[21] Tang H , Zeng L , Wang J , Zhang X , Ruan Q , Wang J , Cui S , Yang D . Reversal of 5-fluorouracil resistance by EGCG is mediate by inactivation of TFAP2A/VEGF signaling pathway and down-regulation of MDR-1 and P-gp expression in gastric cancer. Oncotarget. 2017; 8:82842–53. 10.18632/oncotarget.20666. 29137307 PMC5669933

[22] La X , Zhang L , Li Z , Li H , Yang Y . (-)-Epigallocatechin Gallate (EGCG) Enhances the Sensitivity of Colorectal Cancer Cells to 5-FU by Inhibiting GRP78/NF-κB/miR-155-5p/MDR1 Pathway. J Agric Food Chem. 2019; 67:2510–18. 10.1021/acs.jafc.8b06665. 30741544

[23] Vigasin AA , Slanina Z . Molecular Complexes in Earth’s, Planetary, Cometary and Interstellar Atmospheres. Singapore: World Scientific Publishing Co. 1998. 10.1142/3544.

[24] Higgs PG . Chemical Evolution and the Evolutionary Definition of Life. J Mol Evol. 2017; 84:225–35. 10.1007/s00239-017-9799-3. 28664404

[25] Lehn JM . Supramolecular chemistry. Science. 1993; 260:1762–63. 10.1126/science.8511582. 8511582

[26] Sahoo P . Molecular recognition of caffeine in solution and solid state. Bioorg Chem. 2015; 58:26–47. 10.1016/j.bioorg.2014.11.002. 25462624

[27] Mattoli L , Mercati V , Burico M , Bedont S , Porchia M , Tisato F , D’Aronco S , Crotti S , Agostini M , Traldi P . Experimental Evidence of the Presence of Bimolecular Caffeine/Catechin Complexes in Green Tea Extracts. J Nat Prod. 2018; 81:2338–47. 10.1021/acs.jnatprod.8b00168. 30372064

[28] Crotti S , D’Aronco S , Moracci L , Tisato F , Porchia M , Mattoli L , Burico M , Bedont S , Traldi P , Agostini M . Evidence of noncovalent complexes in some natural extracts: Ceylon tea and mate extracts. J Mass Spectrom. 2020; 55:e4459. 10.1002/jms.4459. 31663260

[29] Moracci L , Crotti S , Traldi P , Agostini M . Mass spectrometry in the study of molecular complexes between 5-fluorouracil and catechins. J Mass Spectrom. 2021; 56:e4682. 10.1002/jms.4682. 33448570

[30] Gao S , Hu M . Bioavailability challenges associated with development of anti-cancer phenolics. Mini Rev Med Chem. 2010; 10:550–67. 10.2174/138955710791384081. 20370701 PMC2919492

[31] Manach C , Scalbert A , Morand C , Rémésy C , Jiménez L . Polyphenols: food sources and bioavailability. Am J Clin Nutr. 2004; 79:727–47. 10.1093/ajcn/79.5.727. 15113710

[32] Pérez-Jiménez J , Torres JL . Analysis of nonextractable phenolic compounds in foods: the current state of the art. J Agric Food Chem. 2011; 59:12713–24. 10.1021/jf203372w. 22070088

[33] Nakamura Y , Tsuji S , Tonogai Y . Method for analysis of tannic acid and its metabolites in biological samples: application to tannic acid metabolism in the rat. J Agric Food Chem. 2003; 51:331–39. 10.1021/jf020847+. 12502429

[34] Seeram NP , Lee R , Heber D . Bioavailability of ellagic acid in human plasma after consumption of ellagitannins from pomegranate (Punica granatum L.) juice. Clin Chim Acta. 2004; 348:63–68. 10.1016/j.cccn.2004.04.029. 15369737

[35] Neilson AP , Hopf AS , Cooper BR , Pereira MA , Bomser JA , Ferruzzi MG . Catechin degradation with concurrent formation of homo- and heterocatechin dimers during *in vitro* digestion. J Agric Food Chem. 2007; 55:8941–49. 10.1021/jf071645m. 17924707

[36] Wang R , Zhou W , Jiang X . Reaction kinetics of degradation and epimerization of epigallocatechin gallate (EGCG) in aqueous system over a wide temperature range. J Agric Food Chem. 2008; 56:2694–701. 10.1021/jf0730338. 18361498

[37] Brandon EF , Oomen AG , Rompelberg CJ , Versantvoort CH , van Engelen JG , Sips AJ . Consumer product *in vitro* digestion model: Bioaccessibility of contaminants and its application in risk assessment. Regul Toxicol Pharmacol. 2006; 44:161–71. 10.1016/j.yrtph.2005.10.002. 16337324

[38] Oomen AG , Tolls J , Sips AJ , Van den Hoop MA . Lead speciation in artificial human digestive fluid. Arch Environ Contam Toxicol. 2003; 44:107–15. 10.1007/s00244-002-1225-0. 12434225

[39] Versantvoort CH , Oomen AG , Van de Kamp E , Rompelberg CJ , Sips AJ . Applicability of an *in vitro* digestion model in assessing the bioaccessibility of mycotoxins from food. Food Chem Toxicol. 2005; 43:31–40. 10.1016/j.fct.2004.08.007. 15582193

[40] Peters R , Kramer E , Oomen AG , Rivera ZE , Oegema G , Tromp PC , Fokkink R , Rietveld A , Marvin HJ , Weigel S , Peijnenburg AA , Bouwmeester H . Presence of nano-sized silica during *in vitro* digestion of foods containing silica as a food additive. ACS Nano. 2012; 6:2441–51. 10.1021/nn204728k. 22364219

[41] Sensi F , D’Angelo E , Piccoli M , Pavan P , Mastrotto F , Caliceti P , Biccari A , Corallo D , Urbani L , Fassan M , Spolverato G , Riello P , Pucciarelli S , Agostini M . Recellularized Colorectal Cancer Patient-derived Scaffolds as *in vitro* Pre-clinical 3D Model for Drug Screening. Cancers (Basel). 2020; 12:681. 10.3390/cancers12030681. 32183226 PMC7140024

[42] D’Angelo E , Natarajan D , Sensi F , Ajayi O , Fassan M , Mammano E , Pilati P , Pavan P , Bresolin S , Preziosi M , Miquel R , Zen Y , Chokshi S , et al. Patient-Derived Scaffolds of Colorectal Cancer Metastases as an Organotypic 3D Model of the Liver Metastatic Microenvironment. Cancers (Basel). 2020; 12:364. 10.3390/cancers12020364. 32033473 PMC7072130

[43] Louris JN , Wright LG , Cooks RG , Schoen AE . New Scan Modes Accessed with a Hybrid Mass Spectrometer. Anal Chem. 1985; 57:2918–24. 10.1021/ac00291a039.

[44] Walczak AP , Fokkink R , Peters R , Tromp P , Herrera Rivera ZE , Rietjens IM , Hendriksen PJ , Bouwmeester H . Behaviour of silver nanoparticles and silver ions in an *in vitro* human gastrointestinal digestion model. Nanotoxicology. 2013; 7:1198–210. 10.3109/17435390.2012.726382. 22931191

[45] Schmoll HJ , Van Cutsem E , Stein A , Valentini V , Glimelius B , Haustermans K , Nordlinger B , van de Velde CJ , Balmana J , Regula J , Nagtegaal ID , Beets-Tan RG , Arnold D , et al. ESMO Consensus Guidelines for management of patients with colon and rectal cancer. a personalized approach to clinical decision making. Ann Oncol. 2012; 23:2479–516. 10.1093/annonc/mds236. 23012255

[46] Hu F , Wei F , Wang Y , Wu B , Fang Y , Xiong B . EGCG synergizes the therapeutic effect of cisplatin and oxaliplatin through autophagic pathway in human colorectal cancer cells. J Pharmacol Sci. 2015; 128:27–34. 10.1016/j.jphs.2015.04.003. 26003085

[47] Deepa P , Kolandaivel P , Senthilkumar K . Interactions of anticancer drugs with usual and mismatch base pairs - density functional theory studies. Biophys Chem. 2008; 136:50–58. 10.1016/j.bpc.2008.04.007. 18501496

[48] Yang CS , Chung JY , Yang G , Chhabra SK , Lee MJ . Tea and tea polyphenols in cancer prevention. J Nutr. 2000 (Suppl 2); 130:472S–78S. 10.1093/jn/130.2.472S. 10721932

[49] Hong J , Lu H , Meng X , Ryu JH , Hara Y , Yang CS . Stability, cellular uptake, biotransformation, and efflux of tea polyphenol (-)-epigallocatechin-3-gallate in HT-29 human colon adenocarcinoma cells. Cancer Res. 2002; 62:7241–46. 12499265

[50] Wielińska J , Nowacki A , Liberek B . 5-Fluorouracil-Complete Insight into Its Neutral and Ionised Forms. Molecules. 2019; 24:3683. 10.3390/molecules24203683. 31614932 PMC6832121

[51] Krook MA , Hagerman AE . Stability of Polyphenols Epigallocatechin Gallate and Pentagalloyl Glucose in a Simulated Digestive System. Food Res Int. 2012; 49:112–16. 10.1016/j.foodres.2012.08.004. 23028206 PMC3460640

[52] Kararli TT . Comparison of the gastrointestinal anatomy, physiology, and biochemistry of humans and commonly used laboratory animals. Biopharm Drug Dispos. 1995; 16:351–80. 10.1002/bdd.2510160502. 8527686

